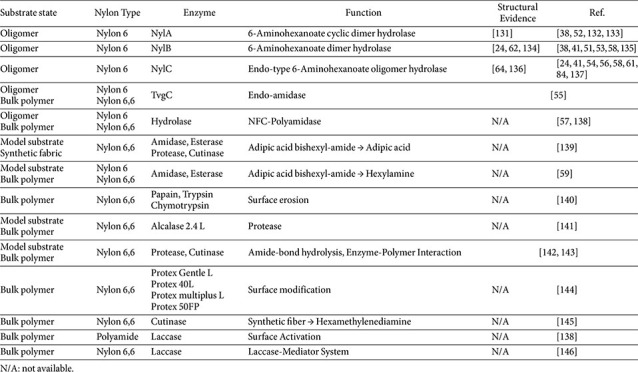# Microbial and Enzymatic Degradation of Nylon: Mechanisms, Diversity, and Biotechnological Application

**DOI:** 10.4014/jmb.2604.04057

**Published:** 2026-05-25

**Authors:** Sun-Yong Jung, Min-Seo Kim, Seung-Do Yun, Hyeoncheol Francis Son, Soo-Jin Yeom

**Affiliations:** 1 Department of Biological Sciences and Biotechnology, Graduate School, Chonnam National University, Gwangju 61186, Republic of Korea; 2 School of Biological Sciences and Technology, Chonnam National University, Gwangju 61186, Republic of Korea; 3 Institute of Synthetic Biology for Carbon Neutralization, Chonnam National University, Gwangju 61186, Republic of Korea; 4 KAIST InnoCORE Artificial Cell Initiative, Korea Advanced Institute of Science and Technology (KAIST), Daejeon 34141, Republic of Korea

**Keywords:** Nylon biodegradation, Microbial diversity, Enzymatic strategies, Nylon upcycling, Biochemistry-informed approaches

## Abstract

Nylon is one of the earliest widely used synthetic polymers based on high crystalline and wear resistance, which make it extensive applications in the automobile, clothing, and consumable industries. The inherent chemical and structural stability of Nylon renders highly recalcitrant to biological degradation, contributing to its accumulation in diverse environments. Nylon biodegradation has therefore attracted considerable attention as a potential route to mitigate environmental persistence and support sustainable recycling and upcycling strategies. This review offers a compendium of Nylon biodegradation, highlights the limitations of current research, and proposes a biochemistry-informed framework that links microbial and enzymatic mechanisms with recycling/upcycling strategies. We highlight five key elements, microbial diversity, enzymatic strategies, Nylon upcycling, future challenges, and biochemistry-informed approaches for Nylon biodegradation. We emphasize the necessity for molecular-level perspective into the relationship between Nylon and Nylon-degrading enzymes. Introducing a molecular perspective into Nylon biodegradation makes the field construct a sustainable Nylon recycling system beyond indirect Nylon-degrading indicators. Also, it enables the rational design of efficient and sustainable Nylon recycling systems. By adopting and constructing system-level framework, this review aims to guide performance enhancement and scalability in further Nylon biodegradation research.

## 1. Introduction

**Fig. 1:**
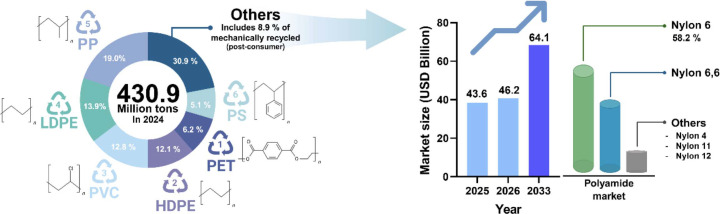
Trends in global Nylon market demand [124-126]. As of 2024, various forms of olefinic and non-olefinic plastics are being utilized industrially. Among them, polyamide, a representative non-olefinic plastic also known as nylon, is widely used in various fields such as textiles and engineering materials due to its excellent heat resistance and wear resistance. In particular, Nylon 6 and Nylon 6,6 lead the market, with Nylon 6 accounting for approximately 58.2% of the total polyamide market. The global market size is projected to grow from approximately $43.6 billion in 2025 to approximately $64.1 billion in 2033.

Polyamides, commonly referred to as Nylons, are polymeric materials that have been widely used across modern industries since their commercialization in the 1930s [1, 2] (Fig. 1). Their extensive use arises from their high mechanical strength, abrasion resistance, thermal stability, and chemical robustness, enabling applications in fibers, plastics, and engineering materials [3, 4]. Among the diverse classes of polyamides, Nylon 6 (PA6), Nylon 6,6 (PA6,6), and Nylon 12 (PA12) represent most of the global production and usage (Fig. 1).

Nylon 6 is synthesized via ring-opening polymerization of ε-caprolactam and is widely used in fibers, packaging materials, fishing nets, and industrial components due to its favorable melt processability and abrasion resistance [5]. Nylon 6,6, a condensation polymer derived from hexamethylenediamine and adipic acid, exhibits higher melting temperature and crystallinity, leading to its superior mechanical strength and thermal resistance in automotive and high-performance engineering applications [6]. In contrast, Nylon 12 contains extended aliphatic segments that reduce intermolecular hydrogen bonding density, resulting in lower moisture uptake and enhanced flexibility. These properties support its use in fuel lines, cable insulation, and medical and electronic devices [7]. Recent advances in bio-based synthetic routes for Nylon 12 monomers have further expanded interest in this polymer from a sustainability standpoint [8, 9].

Despite their extensive use, Nylons remain inadequately incorporated into circular material streams. Post-consumer Nylon waste is predominantly managed through incineration or landfilling, and reported recycling rates remain low [10]. Specifically, 98% of total amount of Nylon production (5.58 million tons) in 2019 is synthesized by the utilization of new resources, while only 2% comes from recycling processes [3]. In marine environments, abandoned Nylon persists over extended timescales and undergoes gradual fragmentation, contributing to the accumulation of microplastics that accumulate throughout marine food webs [11]. In terrestrial systems, Nylon microplastics have been detected in wastewater facilities, landfill leachates, and agricultural soils, prompting sustained concern regarding their long-term ecological impacts [12, 13].

In contrast to polyethylene terephthalate and polyolefins, Nylon has received comparatively limited attention in studies of biological polymer degradation [14]. This imbalance reflects both the intrinsic chemical and structural stability of polyamides and the historical perception on chemical recycling as the primary remediation strategy [15, 16]. Despite these limitations, enzyme-mediated Nylon degradation has attracted interest as a complementary approach to chemical recycling, offering potential advantages such as lower energy requirements, selective recovery of monomeric or oligomeric products, and compatibility with mixed plastic waste streams [17].

The pronounced recalcitrance of Nylon toward biological degradation originates from its polymeric structural characteristics. Although amide bonds are formally analogous to peptide bonds, the combination of high molecular weight, substantial crystallinity (typically 30–50%), and dense interchain hydrogen-bonding networks imposes severe steric and diffusional constraints on enzyme access [18]. Consequently, enzymatic hydrolysis is generally restricted to amorphous regions or polymer surface, yielding minimal degradation to a polymer scale [19, 20]. Furthermore, Nylon is a fully synthetic material without a natural analogue, suggesting that insufficient evolutionary opportunity has existed for the emergence of highly efficient Nylon-degrading enzymes [21, 22].

To date, most reported studies on Nylon degradation have focused on NylA, NylB, and NylC family enzymes that act on low-molecular-weight oligomers derived from Nylon 6, whereas effective degradation of Nylon in the form of high-molecular-weight film or fiber form remains rarely documented [23-25].

Current research is characterized by clear limitations, including the limited availability of enzymes with high catalytic activity and stability, challenges related to substrate accessibility, insufficient understanding of polymer-level degradation mechanisms, reliance on indirect degradation indicators, and the absence of evaluation under industrially relevant process conditions [26].

In this review, we present a systematic overview of microbial and enzymatic studies on Nylon degradation, with a particular focus on Nylon 6 and Nylon 6,6. Rather than simply cataloging Nylon-degrading microorganisms and enzymes, we organize the literature in relation to the mechanistic constraints that have limited progress in Nylon biodegradation, including restricted enzyme access to polymer chains and the challenge of directly verifying amide-bond cleavage. We further discuss how enzyme-level characterization and product-based analyses can strengthen the mechanistic basis of Nylon biodegradation research and support their translation into recycling and upcycling applications. Finally, we outline future directions for enzyme engineering and biotechnological development toward sustainable Nylon recycling.

## Microbial Diversity Involved in Nylon Degradation

There is research against Nylon-biodegradation, which can be classified according to type of Nylon, Nylon conformation, microorganisms, bacterial species, diverse natural environments, experimental conditions (temperature and pH range used in incubation), and analytical methods. In this section, we primarily organize the literature by Nylon type, including Nylon 4, Nylon 6, Nylon 6,6, and Nylon 12, because the chemical structure and physical form of the substrate critically determine degradation outcomes. Also, we dealt with various Nylon material forms such as powder (P), film (F), sheets (S), fiber (Fib), and pellet (Pe). Additionally, we summarize the bacterial or fungal genera and experimental conditions (Table 1). There are several analyses to verify Nylon-degrading ability; these methods include BOD (Biochemical Oxygen Demand), TOC (Total Organic Carbon), growth curve, and weight loss for verifying how much plastic was decomposed by microbes; DSC (Differential Scanning Calorimetry) and TGA (Thermogravimetric Analysis) for analyzing the changed properties of Nylon; FT-IR (Fourier Transform Infrared Spectroscopy), NMR (Nuclear Magnetic Resonance), MALDI-TOF (Matrix-Assisted Laser Desorption/Ionization – Time-of-Flight) and TLC (Thin-layer chromatography) for analyzing the changes of functional groups; SEC (Size-Exclusion Chromatography) and Intrinsic viscosity for finding variation of polymer chain property; SEM (Scanning Electron Microscopy) and TEM (Transmission Electron Microscopy) for morphological comparison; HPLC (High Performance Liquid Chromatography) for qualifying low-molecular weight products. However, the degradation efficiencies reported across the different studies should not be regarded as directly comparable, because differences in substrate form, incubation conditions, and analytical methods can substantially affect the observed degradation outcomes.

### Literature-Based Comparison of Microbial Degradation of Nylon

**Nylon 4**. Three microorganisms (*Stenotrophomonas* sp. KT-1 [27], *Fusarium* sp. KT-2 [27], and *Aestuariibacter halophilus* MND-1 [28]) were used to determine whether the polymer is available for microbial metabolism using the BOD test. When BOD was measured using synthetic Nylon 4 powder, *Stenotrophomonas* sp. KT-1 and *Fusarium* sp. KT-2 showed BOD values of approximately 34% and 27%, Respectively, after 7 days of incubation. *Aestuariibacter halophilus* MND-1 showed a BOD value of approximately 50% after 12 days of incubation. It should be recognized that degradation efficiencies reported in various studies are highly dependent on experimental variables such as incubation time, pretreatment, particle size, and analytical parameters, making direct quantitative comparisons impossible. *Agromyces* sp. NR4 and *Pseudomonas* sp. strain ND-10 showed reducing the weight of Nylon 4 by approximately 65% after 15 days and 0.5% after 14 days, respectively.

**Nylon 6**. Seven microorganisms (five bacteria; *Bacillus cereus* [29], *Vibrio furnisii* [29], *Bacillus sphericus* [29], *Brevundimonas vesicularis* [29], and *Pseudomonas aeruginosa* HE858284 [30] and two fungi; *Phanerochaete chrysosporium* MZKIB223 [31] and *Aspergillus niger* strain AF3 [32]) were tested using intrinsic viscosity analysis to determine whether the polymer decomposed into lower molecular weight compounds. Among seven microbes, five bacteria (*Bacillus cereus* [29], *Vibrio furnisii* [29], *Bacillus sphericus* [29], *Brevundimonas vesicularis* [29], and *Pseudomonas aeruginosa* HE858284 [30]) were compared to determine the strain with the optimal degrading ability of Nylon 6. A 31% decrease in the viscosity of Nylon 6 was observed after 3 months of incubation. Intrinsic viscosity measurements revealed that *Bjerkandera adusta* induced oxidative chain cleavage across the backbone of Nylon 6 after 60 days of incubation, whereas *Aspergillus niger* strain AF3 seemed more consistent with localized hydrolytic cleavage after 90 days of incubation. Weight loss of Nylon 6 was measured using four bacteria (*Bacillus pallidus* strain 26 [33], *Lysinibacillus* sp. [34], *Alcaligenes faecalis* [34], and *Enterococcus faecalis* [34]). The weight loss of Nylon 6 indicated that *Alcaligenes faecalis* was degraded to 27% of the polymer after 48 days of incubation.

**Nylon 6,6.** Five bacteria (*Bacillus cereus* [29], *Vibrio furnisii* [29], *Bacillus sphericus* [29], *Brevundimonas vesicularis* [29], and Brevibacillus brevis [35]) were compared using viscometry and weight loss, and the best resolution of Nylon 6,6 was Brevibacillus brevis after 35 days of incubation. Among the other four bacteria, *Bacillus cereus* seemed the best resolution of Nylon 6,6, reporting around 7 to 8% of Nylon 6,6 biodegradation after 35 days of incubation.

**Nylon 12.** *Bacillus pallidus* strain 26 [33] was confirmed through TOC and bacterial growth curve. The weight loss of Nylon 12 through intrinsic viscometry was measured to 1 g of decrease of treated sample compared to control after 20 days incubation. To the best of our knowledge, reports of Nylon 12 biodegradation are limited, with *Bacillus pallidus* strain 26 being the only strain described so far.

### Limitations of the Taxonomic Approach in Nylon Biodegradation

Nylon degradation ability is not limited to specific microbial species or strains but is distributed across diverse microorganisms, implying a fundamental limitation in predicting Nylon-degrading microorganisms based solely on taxonomic information [36, 37]. Nevertheless, the fact that diverse, phylogenetically unrelated microorganisms exhibit Nylon-degrading ability suggests that Nylon degradation is not an inherent characteristic of specific species or taxa [38]. This underscores the limitations of predicting or screening Nylon-degrading microorganisms solely on taxonomic criteria and emphasizes the need for a molecular and functional understanding of the enzymes and metabolic pathways directly involved in Nylon degradation [36, 37, 39]. Therefore, future research should focus on the structural and functional characteristics and mechanisms of enzymes directly involved in each type of Nylon degradation, rather than on microbial classification [24, 38, 40]. Furthermore, the discovery and optimizing process of key enzymes responsible for Nylon degradation in microorganisms will be required.

## Enzymatic Mechanisms of Nylon Degradation

Nylon is characterized by strong amide bonds and a high degree of crystallinity, which limit enzymatic access to polymer chains and confine degradation reactions largely to the polymer surface [41, 42]. Because of these structural features, enzymatic studies using bulk materials like powder, fiber, and film primarily capture surface-confined reactions rather than bulk degradation throughout the polymer. In parallel, Nylon oligomers or synthetic model substrates have been employed to investigate amide bond hydrolysis under more accessible conditions. The following sections summarize the enzymatic mechanisms of Nylon degradation based on representative enzymes and highlight the complementary insights provided by surface-confined reactions on bulk polymers and cleavage reactions on oligomeric substrates.

### Enzymes Targeting Bulk Nylon Polymers

The first line of research on enzymatic Nylon degradation has focused on evaluating enzyme activity directly on bulk Nylon substrates, including powders, fibers, and films. These studies were primarily designed to assess whether enzymes can interact with intact Nylon polymers under conditions that reflect the physical constraints imposed by high molecular weight and crystallinity. As summarized in Table 2, Enzymes —such as amidase, cutinase and oxidative enzymes including laccases—have predominantly been evaluated in the context of bulk Nylon substrates. Among these enzymes, polyamidases and related amidases are most directly associated with hydrolytic cleavage of amide bonds in Nylon-related substrates, whereas cutinases and proteases have mainly been investigated for their capacity to modify Nylon surfaces or hydrolyze accessible amide-containing model compounds. Oxidative enzymes such as laccases operate through a distinct mechanism, promoting surface oxidation or activation rather than direct hydrolysis of the polyamide backbone. Thus, enzymes evaluated against bulk Nylon substrates differ not only in enzyme class but also in the type of reaction they mediate, ranging from hydrolytic cleavage of accessible amide bonds to surface functionalization or oxidative activation. Across reported cases, enzymes tested against bulk Nylon substrates generally exhibit limited reactivity [43]. Rather than extensive depolymerization, enzymatic treatment typically results in modest surface-level changes—consistent with the bulk polymer cases summarized in Table 2—such as alterations in surface morphology, partial erosion, or the release of small amounts of soluble oligomeric products [44]. These observations indicate that enzymatic reactions are largely confined to surface-accessible regions, highlighting substrate accessibility as a key determinant of reactivity [45, 46]. Even enzymes capable of hydrolyzing amide bonds in soluble substrates show markedly reduced activity when applied to crystalline Nylon polymers [25], suggesting that the limited degradation observed in bulk systems arises from restricted exposure of susceptible bonds rather than from an inherent inability to catalyze amide bond cleavage. Therefore, enzymes acting on bulk Nylon polymers are best understood as mediators of surface-confined reactions, providing insight into enzyme–polymer interactions at the solid–liquid interface rather than into the intrinsic chemistry of amide bond hydrolysis [47]. While such enzymes alone are insufficient to achieve efficient Nylon depolymerization, their surface-modifying activity may facilitate the initial formation or release of low-molecular-weight fragments, which can subsequently serve as substrates for oligomer-specific enzymes [48]. In many cases, improving the enzymatic degradation of Nylon often requires pretreatment strategies, such as reducing crystallinity or increasing surface area, which may enhance substrate accessibility. This limitation has motivated complementary studies using Nylon oligomers and synthetic model substrates, which bypass physical accessibility constraints and enable detailed investigation of amide bond cleavage mechanisms.

### Enzymes Specific to Nylon Oligomers and Synthetic Model Substrates

In contrast to studies employing bulk Nylon substrates, a second line of research has focused on enzymes acting on Nylon oligomers or synthetic model substrates. This approach circumvents the physical accessibility constraints associated with crystalline polymers and enables direct investigation of amide bond hydrolysis under well-defined conditions [49]. By eliminating steric and diffusional limitations, these substrates allow direct investigation of intrinsic enzymatic mechanisms governing amide bond cleavage, whereas bulk polymer systems are dominated by surface interactions [50, 51]. Nylon oligomers generated during polymer manufacturing, as well as chemically synthesized model compounds—as evident from Table 2—have therefore been widely used to probe the intrinsic catalytic capabilities of Nylon-degrading enzymes. Studies in this category have provided detailed insight into the molecular mechanisms of amide bond cleavage, particularly through characterization of Nyl-family enzymes [25, 52-54]. Within the Nyl-family enzymes, NylA, NylB, and NylC perform distinct biochemical roles in the degradation of Nylon 6-derived oligomers. NylA hydrolyzes the cyclic 6-aminohexanoate dimer, whereas NylB acts predominantly on the linear 6-aminohexanoate dimer to generate 6-aminohexanoate. NylC functions as an endo-type hydrolase that cleaves longer 6-aminohexanoate oligomers into shorter soluble products. Collectively, these enzymes represent an oligomer-processing system rather than direct depolymerization of intact Nylon polymers. This distinction is critical, as efficient hydrolysis of water-soluble oligomers does not necessarily imply comparable activity toward high molecular weight or crystalline Nylon substrates. By using soluble substrates, these investigations have allowed systematic analysis of enzyme kinetics, substrate specificity, and active-site architecture, revealing how enzymes recognize and hydrolyze synthetic amide linkages [55-59]. Structural and mutational studies further demonstrate that efficient hydrolysis of Nylon-derived amide bonds is chemically feasible once steric and diffusional barriers are removed [51, 60-64]. Importantly, enzymes characterized using oligomeric or model substrates typically display catalytic efficiencies that are not observed in bulk polymer systems [65, 66]. This discrepancy highlights a fundamental distinction between chemical reactivity and substrate accessibility in Nylon degradation. Taken together, these two research directions illuminate Nylon-degrading enzymes at different levels. Studies on Nylon polymers reveal how enzymes interact with solid polymer surfaces under physically constrained conditions, while investigations using oligomers and model substrates define the molecular principles underlying amide bond hydrolysis. Viewed collectively, these approaches suggest that Nylon degradation is unlikely to proceed through a single enzymatic step, but rather through a multistage, enzyme-mediated cascade, in which initial surface-limited reactions generate soluble oligomeric intermediates that are subsequently processed by oligomer-specific hydrolases [67].

## Biotechnological and Environmental Applications

Enzymatic and microbial degradation of Nylon polymers leads to the release of monomeric products, which can be recovered, purified, and further valorized through chemical and biological conversion. These degradation products represent potential feedstocks for the chemical, materials, environmental, and biotechnology sectors. This section summarizes the chemical identities and industrial relevance of major Nylon degradation products and discusses integrated biotechnological strategies for their conversion into higher-value compounds.

### Major Degradation Products and Their Industrial Value

**ε-Caprolactam.** ε-Caprolactam is the key monomer of Nylon 6 and enables closed-loop recycling by depolymerizing Nylon 6 waste back to its original monomer, which can be directly re-polymerized without loss of material quality [68]. In practice, Nylon 6 is converted to ε-caprolactam through chemical depolymerization processes such as subcritical water treatment or alkaline hydrolysis, followed by purification steps including distillation or crystallization to obtain polymer-grade monomers [69, 70]. This regenerated ε-caprolactam can be reused in the production of virgin Nylon 6, thereby establishing a circular material flow. Beyond direct recycling, ε-caprolactam also serves as a platform chemical for the synthesis of high-value products, including specialty polyamides, coatings, and functional materials with tailored mechanical and thermal properties [71, 72]. Although enzymatic approaches remain limited at the level of intact polymer films, they hold potential as complementary tools during the purification or post-treatment of partially hydrolyzed products and oligomer-rich streams.

**6-Aminocaproic Acid (6-ACA).** 6-Aminocaproic acid (6-ACA) is a bifunctional C6 intermediate generated through hydrolysis of Nylon 6 or via conversion of adipic acid [73]. In recycling processes, Nylon-derived 6-ACA can be recovered and subsequently converted into ε-caprolactam or directly reused as a precursor for polyamide synthesis, thereby contributing to circular material flows [74]. Beyond its role in polymer recycling, 6-ACA serves as a versatile platform chemical to produce high-value compounds. It can serve as a precursor for polyamide synthesis as well as for pharmaceutical intermediates and specialty chemicals. Notably, enzyme cascade reactions combining carboxylic acid reductases and transaminases have enabled efficient biocatalytic conversion of adipic acid to 6-ACA, demonstrating the feasibility of biologically upgrading Nylon-derived intermediates into value-added products [75].

**Adipic Acid and Hexaxmethylenediamine (HMDA).** Hydrolysis of Nylon 6,6 yields adipic acid and hexamethylenediamine, both of which can be recovered as monomeric products and directly reused for Nylon re-polymerization, thereby enabling closed-loop recycling of Nylon 6,6 [76-78]. In practice, depolymerized streams can be separated and purified to obtain polymer-grade adipic acid and HMDA, which are subsequently reintroduced into polycondensation processes for the production of virgin Nylon materials [6]. Beyond direct recycling, adipic acid and HMDA also serve as versatile platform chemicals for the synthesis of high-value products. Adipic acid can be further converted into plasticizers, polyesters, and specialty polymers, while HMDA is widely used in the production of coatings, adhesives, curing agents, and high-performance polyamides. Recent advances in biocatalytic cascades and engineered microbial pathways have further demonstrated the potential to convert these Nylon-derived intermediates into value-added chemicals, offering sustainable alternatives to conventional petroleum-based production routes. [79-81].

**Oligomeric Mixtures.** Incomplete depolymerization of Nylon polymers often results in complex mixtures of linear and cyclic oligomers, which complicate monomer recovery and purification [82]. In recycling processes, these oligomeric species must be removed or further converted to improve monomer yield and product purity. Enzyme-based strategies, including thermostable lactamases and oligomer-specific hydrolases, have been explored to selectively hydrolyze these oligomers into monomeric products or to remove them as impurities, thereby enhancing the efficiency of downstream separation and purification steps [83, 84]. In this context, enzymatic treatment of oligomeric mixtures can contribute to improving overall process efficiency and enabling higher-quality recycled monomers for subsequent material production.

### Upcycling Strategies for Nylon Degradation Products

Beyond monomer recovery, increasing attention has been directed toward converting Nylon degradation products into materials with higher functional value [85, 86]. Integrated chemical–biocatalytic processes, in which chemical depolymerization is used to improve substrate accessibility followed by enzymatic or microbial conversion, are particularly attractive due to their applicability to mixed or contaminated waste streams [87, 88]. For example, microbial fermentation of hydrolysates derived from mixed polyamide fibers has enabled the production of biopolymers such as polyhydroxyalkanoates and bacterial cellulose, demonstrating the feasibility of transforming Nylon waste into functional biomaterials [89-91]. Future upcycling strategies must consider subsequent metabolic transformations following the recovery of Nylon-derived monomers. Since Nylon 6, Nylon 6,6, and Nylon 4 each produce different monomer products, separate downstream transformation pathways must be designed for each polymer type. 6-ACA derived from Nylon 6 can be linked to the caprolactam/6-ACA degradation pathway to produce 6-oxohexanoic acid and adipic acid. The depolymerization of Nylon 6,6 produces adipic acid and hexamethylenediamine. While adipic acid-related metabolic pathways are relatively well understood, the biological transformation of hexamethylenediamine is still understudied. Such approaches highlight the potential of Nylon degradation products as platform substrates for the synthesis of sustainable polymers and specialty chemicals, extending their utility beyond conventional recycling pathways.

### Integrated Biological Systems for Nylon Waste Management

Efficient Nylon waste management is unlikely to rely on a single treatment strategy but instead requires integrated systems that combine pretreatment, enzymatic depolymerization, and microbial conversion [41, 92, 93]. Synthetic microbial consortia have been shown to process complex substrate mixtures more robustly than single strains, and cross-feeding-based metabolism has been reported even in mixed PET–Nylon waste streams [94-97]. In parallel, enzyme cocktails combining endo- and exo-amidases with oligomer-specific enzymes, along with the incorporation of thermostable biocatalysts, are emerging as key strategies for addressing the recalcitrance of semicrystalline Nylon polymers [98, 99].

## Knowledge Gaps and Unresolved Questions

With the recent increase in interest in biodegradation research, numerous Nylon-degrading strains and enzymes have been reported [36, 37, 39]. However, while indirect evidence for microbial degradation has accumulated, the information regarding a clear mechanistic correlation between Nylon degradation observed through various analytical methods and the research of Nylon degradation products remains uncertain [37, 40, 100]. Indeed, many reported studies rely on indicators such as surface erosion, weight loss of Nylon polymer, and SEM or FT-IR changes, which limit the direct comparison and verification of polyamide bond cleavage and product formation [101-103]. Given that Nylon is a polymer composed of amide bonds, soluble degradation products are generated when these bonds are broken during the biodegradation process. Therefore, it is essential to directly identify and verify these degradation products rather than relying solely on surface analysis. As a result, it is more difficult to compare degrading performance at the mechanistic level across different studies, and unexpected variabilities in experimental conditions are likely to limit product reproducibility [104-106]. These shortcomings are further exacerbated by the fact that microbial degradation research relies on complex metabolic pathways that are sensitive to environmental factors such as temperature, pH range, and nutritional availability [39, 107]. Microbes can also activate multiple metabolic pathways during the utilization of Nylon as sole carbon source, making it difficult to clearly trace the pathways and origins of degradation products [22, 38]. In contrast, an approach focused on purified enzymes that directly mediate Nylon biodegradation offers the advantages that simplify the analytical framework, allow for molecular-level analysis for catalytic activity and expect bond cleavage patterns for specific substrates [24, 60, 108, 109]. For instance, whole-genome assembly and analysis of Nylon-degrading microorganisms provide a valuable starting point for identifying potential Nylon-activating enzymes, and in vitro analysis of these Nylonase candidates can elucidate the Nylon degradation mechanism more clearly [36, 110-112]. Furthermore, we identify the molecular determinants contributing to Nylon-biodegrading activity by comprehensively analyzing the structural information and catalytic properties of these enzymes and establish a foundation for rational enzyme design and performance enhancement in the future [109, 113, 114].

### Future Directions of Nylon Biodegradation Based on Synthetic Biology Approaches

Given that the molecular common denominator of Nylon degradation is amide bond cleavage in the Nylon backbone [3, 115, 116], future research needs to expand beyond simple biodegradability assessments to mechanism-based readouts that can directly report amide bond cleavage [22, 117]. Current microorganism-based screening methods often rely on indirect indicators such as growth, mass loss, and surface changes, which pose intrinsic limitations in selectively identifying microorganisms or enzymes with true amide bond cleavage activity [118, 119]. Analytical strategies that directly detect monomers (or oligomers) could provide valuable evidence to verify whether actual amide bond cleavage has occurred [120-122]. As a result, this approach will enable more precise identification of the enzymes responsible for Nylon backbone depolymerization [108]. In particular, the systematic discovery and characterization of Nylon-degrading enzymes will be crucial for advancing the molecular understanding of Nylon biodegradation and developing efficient enzymatic depolymerization strategies [24, 123]. In addition, while genetically encoded biosensors specifically designed to detect Nylon biodegradation products are still limited, recently developed high-throughput product detection assays provide practical pathways for enzyme discovery [61]. In particular, a colorimetric amine detection platform has been used to screen polyamidase activity on Nylon 6 and Nylon 6,6 substrates and to improve Nylon-biodegrading enzyme variants through directed evolution. Overall, the continuous development and integration of these synthetic biology tools is expected to accelerate the discovery, design, and utilization of Nylon degradation biocatalysts, contributing to the establishment of closed-loop Nylon recycling platforms that have not yet been sufficiently studied.

## Figures and Tables

**Table 1:** Microorganisms used in Nylon biodegradation studies.

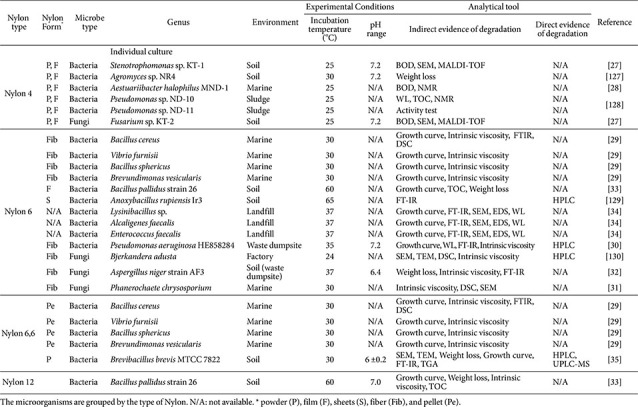

**Table 2:** Classification of Nylon-degrading enzymes based on substrate state and mechanistic features.